# Antimicrobial Resistance, an Update from the Ward: Increased Incidence of New Potential Pathogens and Site of Infection-Specific Antibacterial Resistances

**DOI:** 10.3390/antibiotics9090631

**Published:** 2020-09-22

**Authors:** Irene Stefanini, Martina Boni, Paola Silvaplana, Paola Lovera, Stefania Pelassa, Giuseppe De Renzi, Barbara Mognetti

**Affiliations:** 1 Department of Life Sciences and Systems Biology, University of Turin, Via Accademia Albertina 13, 10123 Turin, Italy; irene.stefanini@unito.it (I.S.); boni.martina12@gmail.com (M.B.); 2 Infectious Risk Prevention Unit, San Luigi Gonzaga University Hospital, Regione Gonzole 10, Orbassano, 10043 Turin, Italy; p.silvaplana@sanluigi.piemonte.it (P.S.); p.lovera@sanluigi.piemonte.it (P.L.); s.pelassa@sanluigi.piemonte.it (S.P.); 3 SCDO Laboratory of Clinical Pathology and Microbiology, San Luigi Gonzaga University Hospital, Regione Gonzole 10, Orbassano, 10043 Turin, Italy; g.derenzi@sanluigi.piemonte.it

**Keywords:** bacterial infection, antimicrobial resistance, nosocomial infections, community-acquired infections

## Abstract

In order to monitor the spread of antimicrobial resistance, the European Union requires hospitals to be equipped with infection control centers. With this aim, we analyzed 1583 bacterial strains isolated from samples of different origin from patients with community-onset and nosocomial infections in a public tertiary University Hospital on the outskirts of Turin, Italy. Statistical analyses of the isolates (source, type) and their antimicrobial resistance (AMR) were performed. The survey revealed infections associated with bacterial species considered as not-commensal and not-pathogenic, hence potentially emerging as new threats for human health. Conversely to the general observation of nosocomial strains being more resistant to antibiotics compared to community-acquired strains, nosocomial strains isolated in this study were more resistant only to 1/42 tested antibiotics (tetracycline). By adopting an ecological approach, we observed that blood infections are associated with the broadest range of species compared to infections affecting other areas and we obtained clear indications on the antibiotics that should be preferred in the treatment of infections at specific body sites. Future investigations carried out on a larger geographical scale will clarify whether these indications are limited to the geographical region investigated over this study, or whether the same trends are visible at national or international level.

## 1. Introduction

The World Health Organization (WHO) defines antibiotic resistance as the ability of a bacterium to grow in the presence of antibiotic concentrations that inhibit the proliferation of bacteria of the same species that are not resistant; this causes the loss of effectiveness of standard treatments and the persistence of the infection [1,2,3]. There is a strong association between antibiotic resistance and antibiotic use, implying that a reduction in unnecessary consumption of antibiotics could affect resistance [4,5,6,7]. According to the 2016–2018 survey, the median consumption of antibiotics in the European Region was 17.9 defined daily dose (DDD) per 1000 inhabitants, and the annual National Observatory on the Use of Drugs survey on drug consumption in Italy confirmed these data, the quantity of antibiotics as DDD per 1000 inhabitants being equal to 18 [8]. Antimicrobials for systemic use represent the third therapeutic category with the highest public expenditure for 2018, equal to 2917 million euros (48.23 euros per capita). The overall positioning of this category is mainly justified by the expenditure deriving from the purchase of these medicines by public health structures (35.16 euros per capita) [9]. The great consumption of antibiotics within hospitals is also strictly correlated to hospital-acquired (HA) infections. The presence of indwelling medical devices such as urinary catheters, feeding tubes and vascular lines is a commonly identified risk factor [10]. Over the last years, bacteria associated with HA infections have been reported to acquire resistance at higher frequency than bacteria associated with community-acquired (CA) infections because of a more severe selective pressure [11]. In order to survey the spread of resistance and to be able to intervene quickly and appropriately by choosing the correct antibiotic to use, it is therefore essential to closely monitor nosocomial and community-acquired infections. The accurate detection of antibiotic-resistant bacteria and selection of the molecule most likely to effectively eliminate them would prevent the evolutionary pressures that accelerate resistance and support antimicrobial stewardship (AMS) approaches; whole-genome sequencing and machine learning techniques [12,13] may nowadays offer the possibility to predict antimicrobial resistance (AMR) outbreak and to rapidly identify targeted treatment of pathogenic bacteria. Although the tools exist, currently the information for effective antibiotics against those infections is widely missing among practitioners and pharmacists both in hospital settings and primary care settings. This knowledge gap sometimes prevents physician– pharmacist collaboration in AMS. An Australian study, on the other hand, demonstrates that when duly informed, general practitioners positively perceive the objectives of AMS, namely reduction of inappropriate use of antimicrobials and health care costs and declare their willingness to participate in AMS training [14]. Gathering information on hospital outbreaks and monitoring the distribution of species-specific and infection-site specific AMR emergence are pivotal instruments for the definition of appropriate and effective clinical treatment of bacterial infections. Aiming at this, our study analyzes bacterial populations isolated from samples of different origin from patients with community-onset and nosocomial infections in a public tertiary University Hospital on the outskirts of Turin, Italy. It emphasizes the diversity of bacteria and their distribution according to isolation source, origin of the infection (nosocomial vs. community-acquired) and resistance to antimicrobials.

## 2. Results

The isolates analyzed over this study derived from different sources are classified as follows: urine catheter positioned in the bladder (BC) or in the ureter (UC), cerebrospinal fluid (CF), blood drawn (DB) and sampled through a catheter (CB). Strains were classified as “hospital-acquired” (HA) if the infection occurred 48 h or more after hospitalization and did not appear to be incubating at the time of admission, otherwise they were considered as “community-acquired” (CA).

### 2.1. Distribution of Isolates

A total of 1583 bacterial isolates were included in this study (Table S1). The 39% of isolates were obtained from CB (*n* = 620), followed by DB and BC (*n* = 486 and 444, respectively 31 and 28% of total isolates). Only 25 and 8 isolates were obtained from UC and CF, respectively (Figure S1). Isolates belonged to 89 species distributed in 33 genera and 24 families (Table S2). Forty-three species were represented by more than 2 isolates (*n* = 1519), 24 species by at least 5 isolates (*n* = 1457), and 63 species by less than 5 isolates (*n* = 126). Twenty-eight species, belonging to 21 genera, were represented by only one isolate (Table S2). The most frequent species were *Escherichia coli* (*n* = 260 isolates), *Staphylococcus epidermidis* (*n* = 239), *Klebsiella pneumoniae* spp. *pneumoniae* (*n* = 154), *Staphylococcus hominis* (*n* = 141), *Staphylococcus aureus* (*n* = 118), *Enterococcus faecalis* (*n* = 101). All the CF isolates were Gram-positive, belonging to the *Staphylococcus* genus (4/6) or to the *Corynebacterium matruchotii* and *Streptococcus pyogenes* group A species. Overall, only six isolates (0.4%), 3 of which from DB and 3 from BC, were identified as *Acinetobacter*. Some species usually not associated with infections were identified: *Staphylococcus intermedius, Staphylococcus lentus, Staphylococcus schleiferi, Ochrobactrum anthropic,* and *Agrobacterium radiobacter* (Table S2). Strains belonging to these species were mainly associated with nosocomial infections (8/9). Eight of these strains were isolated from blood-related specimens (DB or CB) and only one from BC. Only two of these strains, both *S. intermedius*, possibly originated from the same source, as they were identified in patients hospitalized at the same ward and over the same period (Table S2). Four of these isolates were found in co-occurrence with other species isolated from the same patient, namely *K. pneumoniae spp pneumoniae, S. hominis*, and *S. aureus* (Table S2).

### 2.2. Hospital (HA) and Community-Acquired (CA) Infections

**Figure 1:**
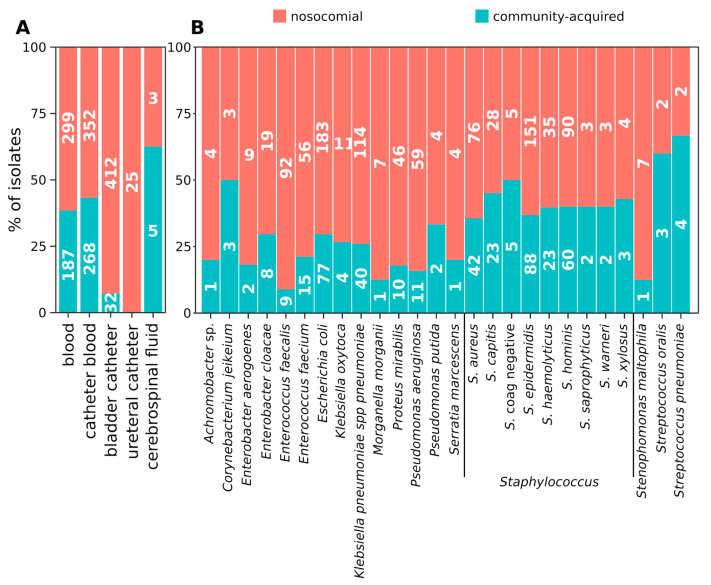
Distribution of nosocomial and community-acquired isolates according to the source of isolation and bacterial species. An isolate was considered “community-acquired” if the infection occurred within the first 48 h after the hospitalization of the patient, “nosocomial” if the infection occurred after 48 h. (**A**) distribution of nosocomial and community-acquired isolates according to the source of isolation. (**B**) distribution of nosocomial and community-acquired isolates according to the bacterial species. Only the bacterial species represented by at least five isolates are represented.

A figure of 69% (*n* = 1091) of the strains was associated with HA infections. To note, while only *E. faecalis* was found to be more likely associated with HA than with CA infections, the other species were equally distributed. CF and BC strains were significantly enriched in HA isolates (Wilcoxon fdr = 0.019 and 0.001, respectively). In fact, all the CF isolates (25/25) and the vast majority of strains isolated from BC (412/444 total isolates) were HA (Figure 1A). Contrarily, only 61.5% of DB isolates (299/486), 56.8% of CB isolates (352/620) and 62.5% of UC isolates (5/8) were HA. It is worth noting that the adopted definition of HA and CA may not be adequate for UC isolates as the timing of the collection of such samples is biased by the surgical time-schedule. Despite none of the most abundantly isolated microbial species including only HA or CA strains, *E. faecalis* strains were more likely to be HA than CA (Wilcoxon fdr = 0.003) (Figure 1B).

**Figure 2:**
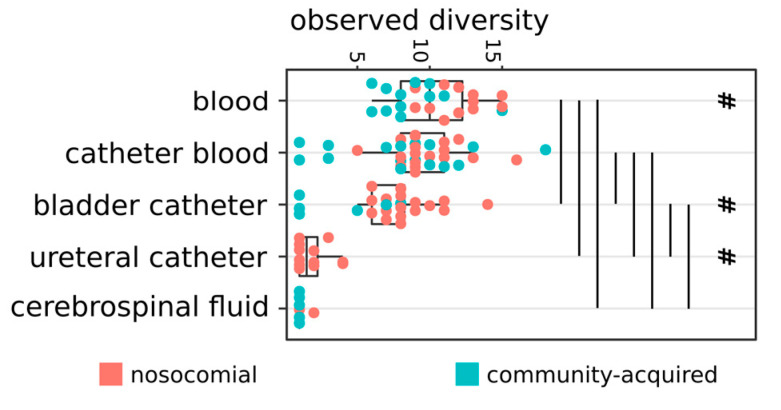
Number of species per group of isolates. Strains isolated at the same time (month and year) from the same type of specimen and recognized as having the same origin (nosocomial or community-acquired) were considered as belonging to the same group. Vertical lines show significant differences among the connected groups of strains (Wilcoxon fdr < 0.05); # = significant differences between the number of species in nosocomial and not-nosocomial groups from the corresponding specimen (Wilcoxon fdr < 0.05).

To note, the majority of isolates belonging to not-commensal nor opportunistic pathogen species, listed in the previous paragraph, was associated with HA infections (Table S3). Notably, all the bacteria isolated from CF were Gram-positive, belonging to the *Staphylococcus* genus (4/6) or to the *Corynebacterium matruchotii* and *Streptococcus pyogenes* group A species. DB strains belonged to a significantly higher number of species compared to strains isolated from the other specimens (Wilcoxon fdr < 0.05, Figure 2). CB strains, in turn, belonged to more species compared to BC, UC, and CF isolates, and BC strains encompassed more species compared to UC and CF specimens (Wilcoxon fdr < 0.05, Figure 2). Nosocomial DB and BC specimens bore more species compared to CA samples (Wilcoxon fdr < 0.05, Figure 2).

### 2.3. Distribution of Species

**Figure 3:**
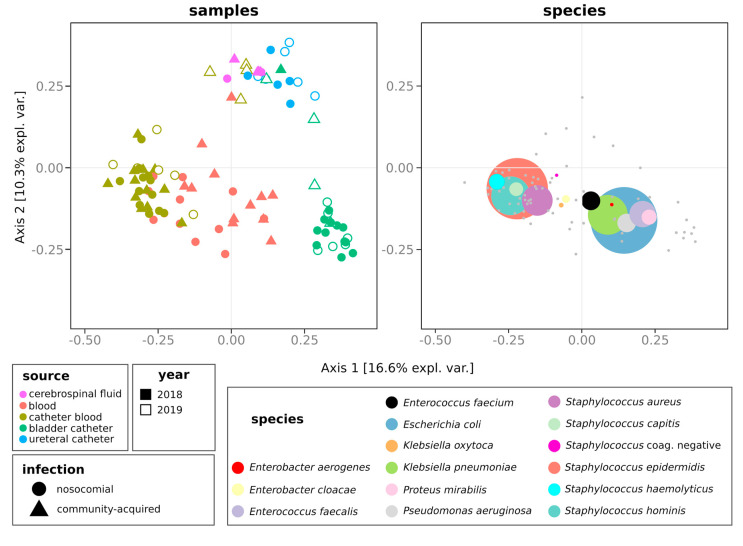
Comparison of the composition of groups of isolates. Distribution of variables over the first two components of the principal coordinate analysis (PCoA) carried out on Jaccard distances. Strains isolated at the same time (month and year) from the same type of specimen (blood, catheter blood, bladder catheter, ureteral catheter, or cerebrospinal fluid) and recognized as having the same origin (nosocomial or community- acquired) were considered as belonging to the same group. Left: first two coordinates of the PCoA. Right: the same PCoA plot with the fifteen most abundant species (showing the highest average relative abundance across the dataset) overlaid as colored points with a size proportional to the mean relative abundance of the taxon across all groups of isolates. Species coordinates were calculated as the weighted average across groups of samples coordinates. Grey dots in the right plot represent the coordinates of species present at lower abundances.

We then compared the composition, in terms of microbial species, of groups of isolates. The profiles of microbial species significantly differed among samples isolated from different sources (permanova fdr = 0.001, Figure 3).

**Figure 4:**
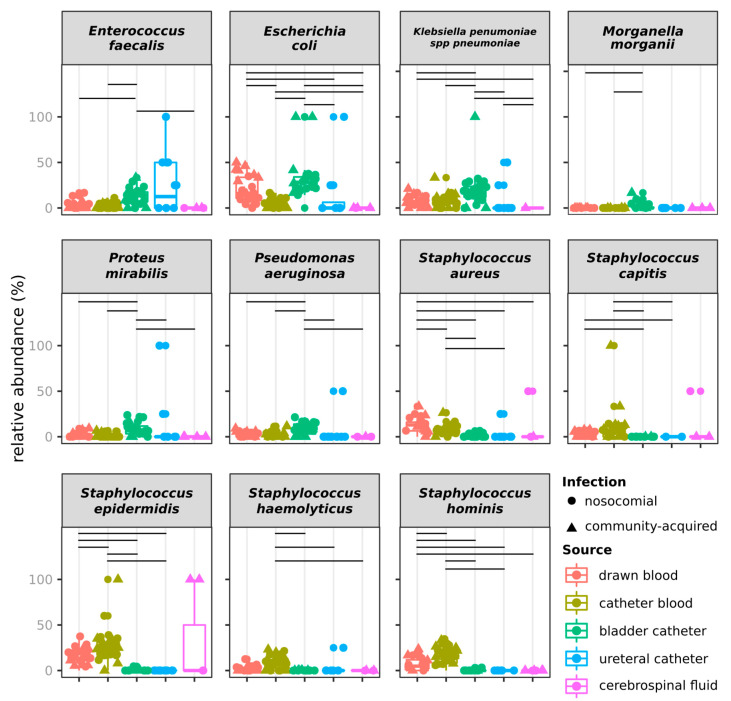
Bacterial species with relative abundances differing among sources of isolation over the whole dataset. Horizontal lines indicate significant differences (Wilcoxon test fdr < 0.05).

Species composition discriminated isolates also according to infection type (permanova fdr = 0.01) and to the year (permanova fdr = 0.002) but not to the month of isolation (permanova fdr = 0.752). Considering that whereas the 2019 strains referred only to the first semester, 2018 strains referred to the whole year, we assessed if the observation of different species composition was confirmed by comparing the same semester of the two years (Figure S2). Even in this case, the species composition discriminated groups of isolates according to the source, the origin of infection, and year, but not according to the month of isolation (permanova fdr > 0.05, Figure S2). None of the species showed different abundances over the two years when using the whole dataset. Contrarily, when focusing on just the first semester of both years, the relative abundance of *Staphylococcus aureus* was higher in 2018 than in 2019 (Figure S3). *E. faecalis, E. coli, K. pneumoniae, M. morganii, Proteus mirabilis,* and *Pseudomonas aeruginosa* were more frequently isolated from BC than from other sources (Welch *t*-test fdr < 0.05, Figure 4).

In addition, *E. coli* was more frequently isolated from DB than from CB, UC, and CF. Similarly, *S. aureus* was more frequently isolated from DB than from any other specimen, and from CB than from BC and UC. *S. epidermidis, S. haemolyticus* and *S. hominis* were more frequently isolated from blood (DB and CB) than from other sources. The same comparison on strains isolated over the first semester of 2018 and 2019 confirmed the results observed on the whole dataset for *E. faecalis, E. coli, P. aeruginosa, S. epidermidis, S. haemolyticus* and *S. hominis* (Figure S3).

### 2.4. Antimicrobial Resistance of Isolates

**Figure 5:**
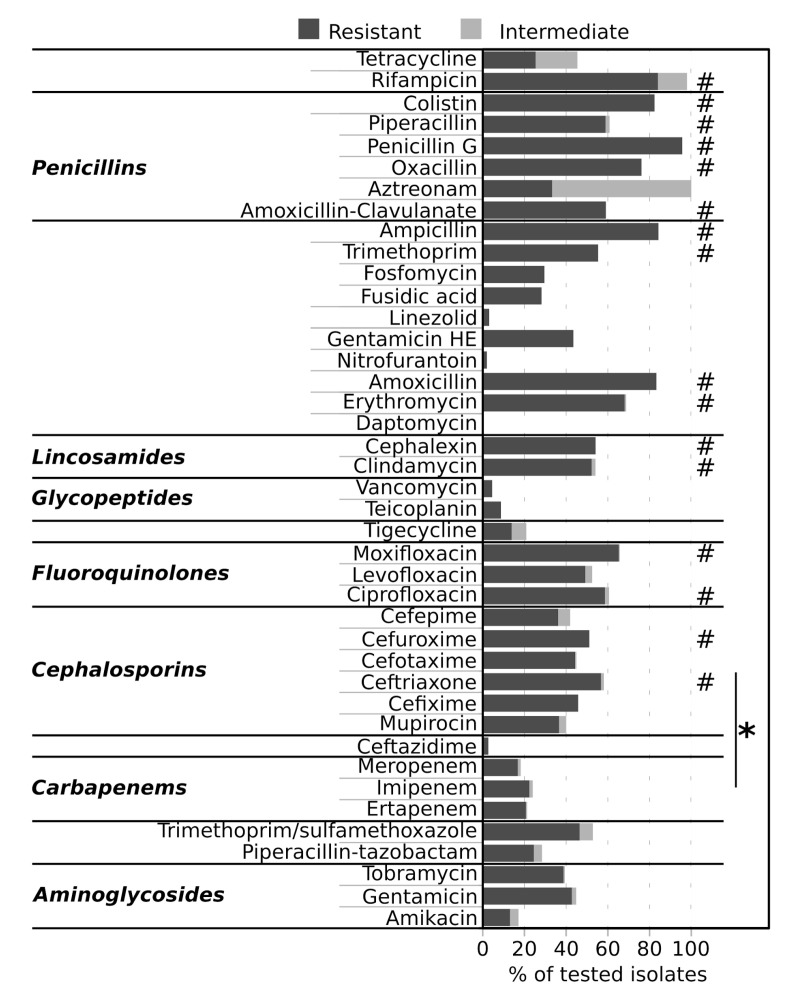
Resistance of isolates to antibiotics. Bars indicate, for each tested antibiotic, the percentage of tested isolates that were resistant or intermediate to the antibiotic. # = two-sided, unpaired Welch *t*-test fdr < 0.05, comparison between antibiotics; * = Wilcoxon fdr < 0.05, comparison between antibiotic classes.

The 9.85% of strains (156 strains) were susceptible to every tested antibiotic (42 total antibiotics), 4 strains (0.25%) were intermediate to all the tested antibiotics, and the 4.74% (75 strains) were resistant to every tested antibiotic. Of the 75 strains belonging to the latter group, 7 were isolated from BC, 25 from DB, and 43 from CB; 39 isolates were CA and 36 were HA; 3 were isolated in 2019 and 72 in 2018 (Table S1). Overall, isolates showed the highest percentages of resistance to ceftriaxone, cefuroxime, ciprofloxacin, moxifloxacin, clindamycin, cephalexin, erythromycin, amoxicillin, trimethoprim, ampicillin, amoxicillin-clavulanate, oxacillin, penicillin G, piperacillin, colistin and rifampicin (Welch fdr < 0.05, Figure 5) and were significantly more resistant to cephalosporins than to carbapenems (Figure 5).

We did not detect significant differences in terms of percentages of resistance to any of the tested antibiotics among commensal, not-commensal, and other bacterial species, with “other” bacterial species being the species that, as described earlier, were not commonly associated with infections nor identified as commensal (Wilcoxon test fdr > 0.05).

**Figure 6:**
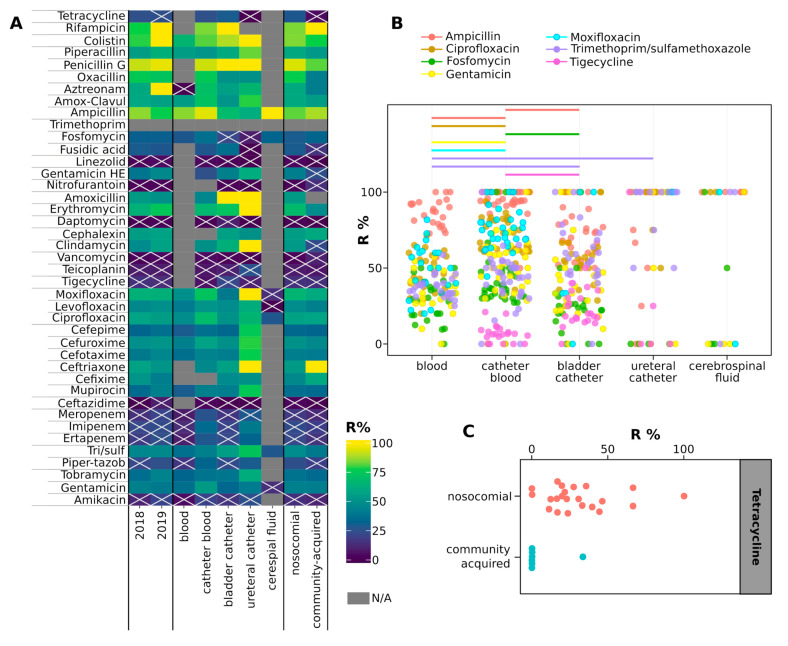
(**A**) Heatmap showing the percentages of resistant strains grouped according to the classification indicated in columns. The color of each cell shows the percentage of strains resistant to the antibiotic listed in rows, with N/A indicating that strains of the corresponding group were not tested with the antibiotic indicated in the row. Crossed cells indicate groups of isolates not significantly more resistant to the antibiotic indicated in the row compared to the rest of the antibiotics (two-sided, unpaired Welch *t*-test fdr > 0.05, comparing the % of strains of the group resistant to the antibiotics to the % of resistances of the group of strains). (**B**) box plots showing the percentages of strains resistant to the listed antibiotics according to the isolation source. Only drugs for which significant differences in resistance percentages have been observed between isolation sources are shown. Horizontal lines indicate significant differences among resistance percentages to the antibiotic indicated with the color (Wilcoxon test fdr < 0.05). (**C**) box plot showing the percentages of strains resistant to the listed antibiotics according to the infection type. In panels B and C, each point represents a group of isolates (strains isolated from the same specimen, in the same year and month, and associated with the same type of infection).

Strains grouped according to the year of isolation, isolation source or infection type showed a lower percentage of resistance to linezolid, ampicillin-sulbactam, daptomycin, vancomycin, teicoplanin, tigecycline, ceftazidime and amikacin than to other antibiotics as detailed in Figure 6A. We could not find differences among the susceptibilities of strains isolated in 2018 and those isolated in 2019 (Table S4). Contrarily, strains associated to HA infections were significantly more resistant than CA-associated strains to tetracycline (Wilcoxon fdr < 0.05, Figure 6C), which was tested only against *Staphylococcus* spp. and *Corynebacterium* spp. It is worth to note, however, that resistance in the other *Staphylococcus* and *Campylobacter* isolates was comparable to the other species (Figure S4). Furthermore, CB strains were more resistant to ampicillin and fosfomycin than BC isolates, and to gentamicin, ciprofloxacin, and moxifloxacin compared to DB isolates, but they were less resistant to ampicillin compared to DB isolates and to tigecycline compared to BC isolates (Wilcoxon fdr < 0.05, Figure 6B). Similarly, strains isolated from DB were more susceptible to trimethoprim/sulfamethoxazole than strains isolated from BC and UC (Wilcoxon fdr < 0.05, Figure 6B). *Achromobacter* spp., *Staphylococcus saprophyticus* and *K. pneumoniae* spp. *pneumoniae* showed higher percentages of resistance than any other species (Figure S4). Contrarily, *E. faecalis, Klebsiella oxytoca, Pseudomonas putida, Serratia marcescens, Staphylococcus warneri, Stenotrophomonas maltophilia* and *Streptococcus pneumoniae* showed lower percentages of resistance to antibiotics than other species (Figure S4).

**Figure 7:**
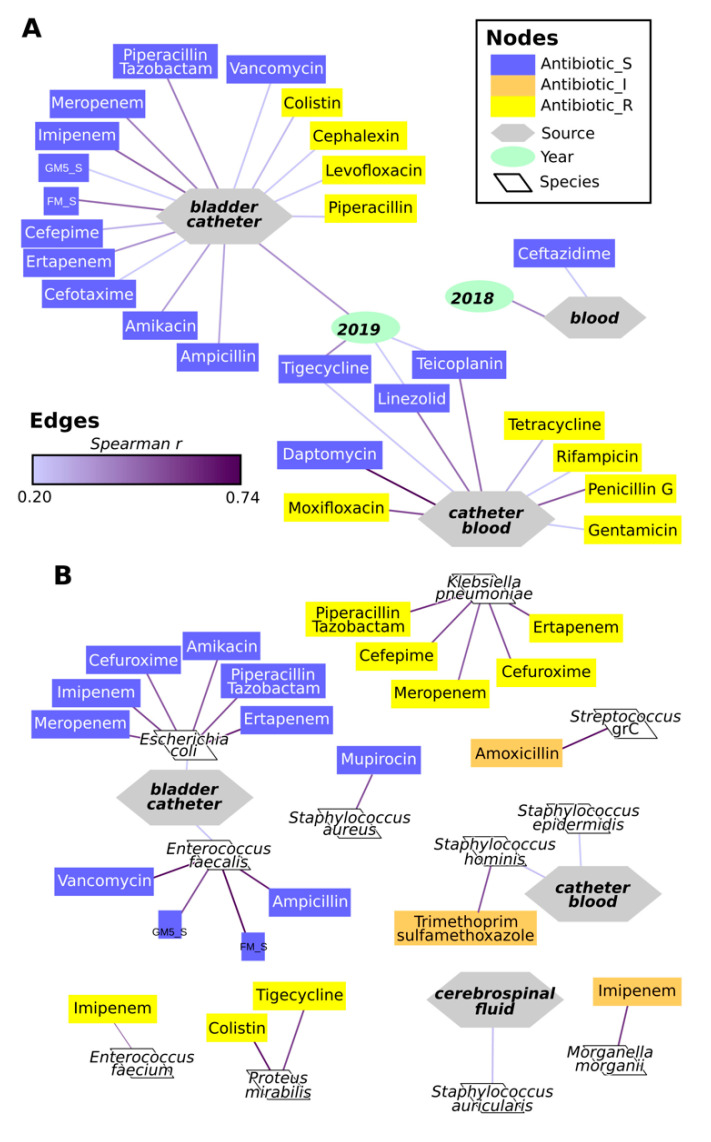
Spearman correlations among year, source of isolation, microbial species, and susceptibility to antibiotics. (**A**) correlations among year and source of isolation and susceptibility to antibiotics. (**B**) correlations among microbial species and sources and year of isolation and susceptibility to antibiotics. Edges indicate the presence of a significant correlation (Spearman correlation r > 0.2 and fdr < 0.05), with the color of the line indicating the correlation r, as indicated in the legend. To simplify the representation, only correlations with r > 0.5 are shown for relations between microbial species and other variables.

The isolation from BC was correlated with resistance to ampicillin and many other antibiotics, and with the susceptibility to colistin, cephalexin, levofloxacin, and piperacillin (Spearman r > 0.2, fdr > 0.05, Figure 7A). Similarly, strains susceptibility to moxifloxacin, tetracycline, rifampicin, penicillin G or gentamicin, or resistance to daptomycin, linezolid, teicoplanin or tigecycline were correlated with isolation from CB. Significant correlations were also found between the year of isolation 2019 and the source BC and resistances to tigecycline, linezolid and teicoplanin (Figure 7A). *E. coli* and *E. faecalis* were significantly correlated with the resistance to specific antibiotics as represented in Figure 7B. Similarly, *S. aureus* was correlated with the resistance to mupirocin; however, it has to be taken into account that this antibiotic was tested on *S. aureus* only (Table S3). Some species were correlated with susceptibility to antibiotics: *E. faecium* with the susceptibility to imipenem, *Proteus mirabilis* to colistin and tigecycline, *K. pneumoniae* spp. *pneumoniae* to ertapenem, cefuroxime, meropenem, cefepime and piperacillin/tazobactam (Figure 7B). Correlations were also found between intermediate susceptibility to amoxicillin and *Streptococcus* spp., to imipenem and *Morganella morganii*, and to trimethoprim-sulfamethoxazole and *Staphylococcus hominis*. Furthermore, *Staphylococcus auricularis* was correlated with isolation from CF, and *Staphylococcus hominis* and *Staphylococcus epidermidis* with isolation from CB (Figure 7B).

## 3. Discussion

The distribution of isolates according to the source of origin shown by our analysis is in accordance with a recent national survey indicating blood as one of the most common sites of infections [15]. The incidence of bacterial species partially mirrored national and international surveys indicating *Staphylococcus spp., E. coli* and *Klebsiella* spp. as the most frequent bacteria [15,16,17]. The analysis of the species, on the other side, highlighted a peculiarity of the population studied: only six isolates (0.4%) were identified as *Acinetobacter*, one of the genera most frequently associated with infections in Italy [15]. This partial discrepancy could be ascribed to either peculiar epidemiology of bacterial infections in Turin or, more likely, to the different extent of the studies. In fact, whereas the national survey investigated 544 cases of sepsis [15], the current study reports 1583 strains not necessarily associated with sepsis. Our study does not include isolates from respiratory tract infections because of a lack of conformity in data acquisition and management with the strains herein analyzed. Further separate studies on this set of isolates has the potential to enhance our understanding of AMR acquisition and spread.

Despite the vast majority of isolates belonging to commensal and opportunistic pathogen species, some species usually not associated with infections were identified, potentially indicating the expansion of the range of pathogenic bacteria due to an increase in the exposure to such species, in the susceptibility of individuals or in the pathogenic potential of these bacteria. The first hypothesis is not likely as humans have been exposed for decades to the potential new pathogens: the listed *Staphylococcus* species are commonly isolated from pigeons, dogs, and cats [18,19,20] and *Agrobacterium radiobacter* is an environmental species. Contrarily, the latter two hypotheses are both plausible, as the very few reports on infections caused by these species in humans have always been observed in patients suffering from debilitating diseases [20]. The higher frequency of hospital-acquired than community-acquired infections observed in this study could also be the result of several factors. First of all, the characteristics of the patients, already proposed to play a major role in determining the probability of insurgence of nosocomial infections [15]. Second, different care from family doctors may also be relevant, with the number of observed CA infections being reduced because patients are treated by the family doctor and not hospitalized.

The prevalence of HA over CA origin observed here for BC and CF isolates was previously observed [21] and ascribed to the compresence of immune system impairment or medical procedures in hospitalized patients making the individual more susceptible to this type of infection [22]. Notably, despite recent reports highlighting an increase of infections of the central nervous system caused by Gram-negative bacteria [22], all the CF bacteria analyzed here were Gram-positive. When considering the variability among isolation sources, our results indicate that multiple species can be isolated from DB, BC and UC in HA infections, and a lower number of species are associated with CA infections at these districts.

The absence of significant differences in the abundance of strains isolated over different months suggests that no outbreaks have occurred in the studied hospital. In fact, we would otherwise have observed a sudden increase in the number of strains belonging to the outbreaking species, even without considering every single analyzed ward. When examining the differences among groups of isolates, the observed high species richness of DB isolates and the different abundance of a few bacterial species among districts not only confirm the known list of bacterial species causing CA infections but also highlight a differential distribution of pathogenic species over different districts.

A major problem in antimicrobial therapy is represented by the diffusion of AMR, and our study confirms this trend by evidencing 75/156 strains resistant to every tested antibiotic. About half of these strains were isolated from CA and a half from HA, contrasting with the hypothesis that HA bacteria are more prone to acquire antibiotic resistance because of a more severe selective pressure [11]. Among resistant bacteria, only *Staphylococcus saprophyticus* isolates showed resistance to a larger number of antibiotics compared to the others, confirming recent reports indicating the insurgence of resistance in this species [23]. It can be excluded that the observed high resistance of HA isolates to tetracycline is misled by the subset of tested species, because they are not significantly more resistant than other species, and that it is caused by a prolonged exposure of strains to the antibiotic, because tetracycline is not commonly prescribed in the studied hospital.

In our study, strains isolated from DB were more susceptible to trimethoprim/sulfamethoxazole than strains isolated from other sources, and *Achromobacter* spp., *Staphylococcus saprophyticus* and *Klebsiella pneumoniae* spp. *pneumoniae* showed higher percentages of resistance compared to all the other species. The latter species is already known to be frequently associated with multi-antibiotic resistance and promoting the spread of antibiotic resistance genes [24]. Contrarily, the lower percentages of resistance to antibiotics detected in some species suggest a new reassuring trend, at least in the studied hospital, considering that several of these species have shown multidrug resistance [25].

Together with the observation of higher species richness of CB strains, the correlation between isolation from this site and resistance to several antibiotics (moxifloxacin, tetracycline, rifampicin, penicillin G, and gentamicin) clearly indicates the need for particular strategies to eradicate these infections. Isolates from BC were significantly correlated with two species (*E. coli* and *E. faecalis*), which were in turn correlated with resistance to multiple antibiotics. In this optics, the antimicrobials toward which DB or BC isolates are susceptible should be preferentially adopted to eradicate infections in those districts. On the other hand, the antimicrobials, the resistance of which is correlated with certain isolation sources, should be reserved to treat infections in other districts.

Our study has strengths and limitations. We clearly highlight several insights useful for both the fight against antimicrobial resistance and the improvement of the success of site-specific infections, therefore contributing to provide information for effective treatment. On the other hand, the local dimension of the population explored does not permit general conclusions to be extrapolated that can be globally extended. Moreover, our study does not include isolates from respiratory tract infections or other sources.

## 4. Materials and Methods

### 4.1. Isolation of Strains

The information on the isolates analyzed in this study were collected at the San Luigi Gonzaga Hospital in Turin (Italy) (Tables S1 and S2). The sources of isolation were: urine catheter positioned in the bladder (BC) or in the ureter (UC), cerebrospinal fluid (CF), blood drawn (DB) and sampled through a catheter (CB). Strains were classified as “hospital-acquired” (HA) if the infection occurred 48 h or more after hospitalization and did not appear to be incubating at the time of admission, otherwise they were considered as “community-acquired” (CA) [26]. The study includes strains isolated from samples collected between January 1st, 2018 and June 30th, 2019. All the strains isolated over this period were included in the study. Strains were isolated on selective media (VACUTEST KIMA S.r.L., Arzagrande (PD), Italy; BIOMERIEUX ITALIA S.P.A., Bagno a Ripoli (FI), Italy) and identified according to their growth and metabolic profiles. Both bacterial identification and antibiogram assessment were automatized and standardized by using the BD Phoenix™ hardware (Becton Dickinson Italia S.P.A., Milano, Italy).

### 4.2. Antimicrobial Testing

The set of tested antibiotics was decided on according to the isolate species (Tables S3 and S4). Antibiotic susceptibility was assessed through the Broth microdilution methodology according to ISO and EUCAST. Each isolate was scored as R (Resistant), I (Intermediate), or S (Susceptible) to the antibiotics according to the clinical breakpoints tables v8.0 and v9.0 (for the 2018 and 2019 isolates respectively) defined by EUCAST (Table S4).

### 4.3. Statistical Analyses

For community composition analyses, isolates were grouped as having the same date of isolation (month and year), isolation source, and being HA or CA. The main text clearly reports where this grouping has been used (referred to as “group of isolates”). Alpha diversities (within-sample richness, number of species) were computed using the phyloseq R package [27]. Principal Coordinate analyses were carried out by using the function ordinate() in the phyloseq library on Jaccard distances calculated on the composition of microbial communities in groups of isolates. Permanova (Permutational multivariate analysis of variance) was performed using the Adonis() function of the vegan R package with 999 permutations. Two-sided, unpaired Welch t-statistics were computed using the function mt() in the phyloseq library [27] and the *p*-values were adjusted for multiple comparisons controlling the family-wise Type I error rate (minP procedure) [28]. Unpaired two- samples Mann–Whitney U test (Wilcoxon test) statistics were computed using the function wilcox.test() in the stats library [29] and the *p*-values were adjusted for multiple comparisons by computing false discovery rate (FDR)-adjusted *p*-values using the Benjamini–Hochberg procedure [30]. For each tested antibiotic, resistance percentage was calculated as the number of resistant isolates over the total of isolates tested with the corresponding antibiotic. Spearman correlations were assessed on categorical variables by converting the database into a 0/1 matrix. Multi-level variables were separated. For instance, the isolation source was split into five variables (DB, CB, BC, UC, and CF) coded as 1 if the strain was isolated from the corresponding source or as 0 if the strain was not isolated from that source. The same approach was used for the year of isolation and the type of infection (HA and CA). The profiles of susceptibility to the tested antibiotics were converted to 0/1 vectors. In this case, for each tested antibiotic, three factors were generated (R, I, and S) and each isolate was scored as 1 for the corresponding phenotype and as 0 for the other phenotypes.

## 5. Conclusions

Despite showing a general trend of bacterial species associated with infections and antibiotic resistance complying with national and international surveys, this study highlights some new indications. The most striking new findings range from the observation of an increased incidence of infections caused by not-commensal bacterial species rarely considered pathogenic, to clear indications on the antibiotics that should be preferred in the treatment of infections at specific body sites. Future investigations on a larger geographical scale will clarify whether these indications are limited to the geographical region investigated in this study (Turin, Italy), or whether the same trends are shown at national or international level.

## Supplementary Materials

The following are available online at https://www.mdpi.com/2079-6382/9/9/631/s1, Figure S1: Composition of the dataset of isolates; Figure S2: Comparison of the composition of groups of strains isolated over the first semester of 2018 and 2019; Figure S3: Bacterial species with relative abundances differing among years or sources of isolation over the first semester of 2018 and 2019; Figure S4: Antibiotic resistance per species. Table S1: full metadata on the strains isolated and analyzed in this study; Table S2: Taxonomic classification of strains isolated in this study and clinical characteristics of the species; Table S3: List and details on antibiotics tested in this study; Table S4: Results of statistical tests carried out to compare Antimicrobial Resistance of isolates grouped according to the type of infection, isolation source or year of isolation.
