# Supplementary material for: Antimicrobial Resistance, an Update from the Ward: Increased Incidence of New Potential Pathogens and Site of Infection-Specific Antibacterial Resistances
Source: Antibiotics (Basel). 2020 Sep 22;9(9):631. doi: 10.3390/antibiotics9090631 (PMC7558927; doi:10.3390/antibiotics9090631)

Appendix to

# **Antimicrobial resistance, an update from the ward: increased incidence of new potential pathogens and site of infection-specific antibacterial resistances**

Submitted to: **Antibiotics**

### Irene Stefanini, Martina Boni, Paola Silvaplana, Paola Lovera, Stefania Pelassa, Giuseppe De Renzi, Barbara Mognetti

Corresponding author: Barbara Mognetti

Department of Life Sciences and Systems Biology, University of Turin, Via Accademia Albertina 13. 10123 Turin, Italy

e-mail address: [barbara.mognetti@unito.it](mailto:barbara.mognetti@unito.it)

**Supplementary Figures**

[**Figure S1**](#bookmark=id.tyjcwt)**:** Composition of the dataset of isolates.

[**Figure S**](#bookmark=id.3dy6vkm)**2:** Comparison of the composition of groups of strains isolated over the first semester of 2018 and 2019.

[**Figure S**](#bookmark=id.1t3h5sf)**3:** Bacterial species with relative abundances differing among years or sources of isolation over the first semester of 2018 and 2019.

Figure S4: Antibiotic resistance per species.

**Supplementary Tables**

**Table S1:** full metadata on the strains isolated and analyzed in this study (provided as a separated file, “Table_S1_dataset.xslx”).

**Table S2**: Taxonomic classification of strains isolated in this study and clinical characteristics of the species (provided as a separated file, “Table_S2_species_distribution.xslx”).

**Table S3**: List and details on antibiotics tested in this study (provided as a separated file, “Table_S3_antibiotics.xslx”).

**Table S4**: Results of statistical tests carried out to compare Antimicrobial Resistance of isolates grouped according to the type of infection, isolation source or year of isolation (provided as a separated file, “Table_S4_Resistances_by_groups_tests.xslx”).

**Figure S1**

**Composition of the dataset of isolates.** A- distribution of isolates; B- distribution of species. “others”= all the species with a frequency of isolation lower than 1% in the corresponding source


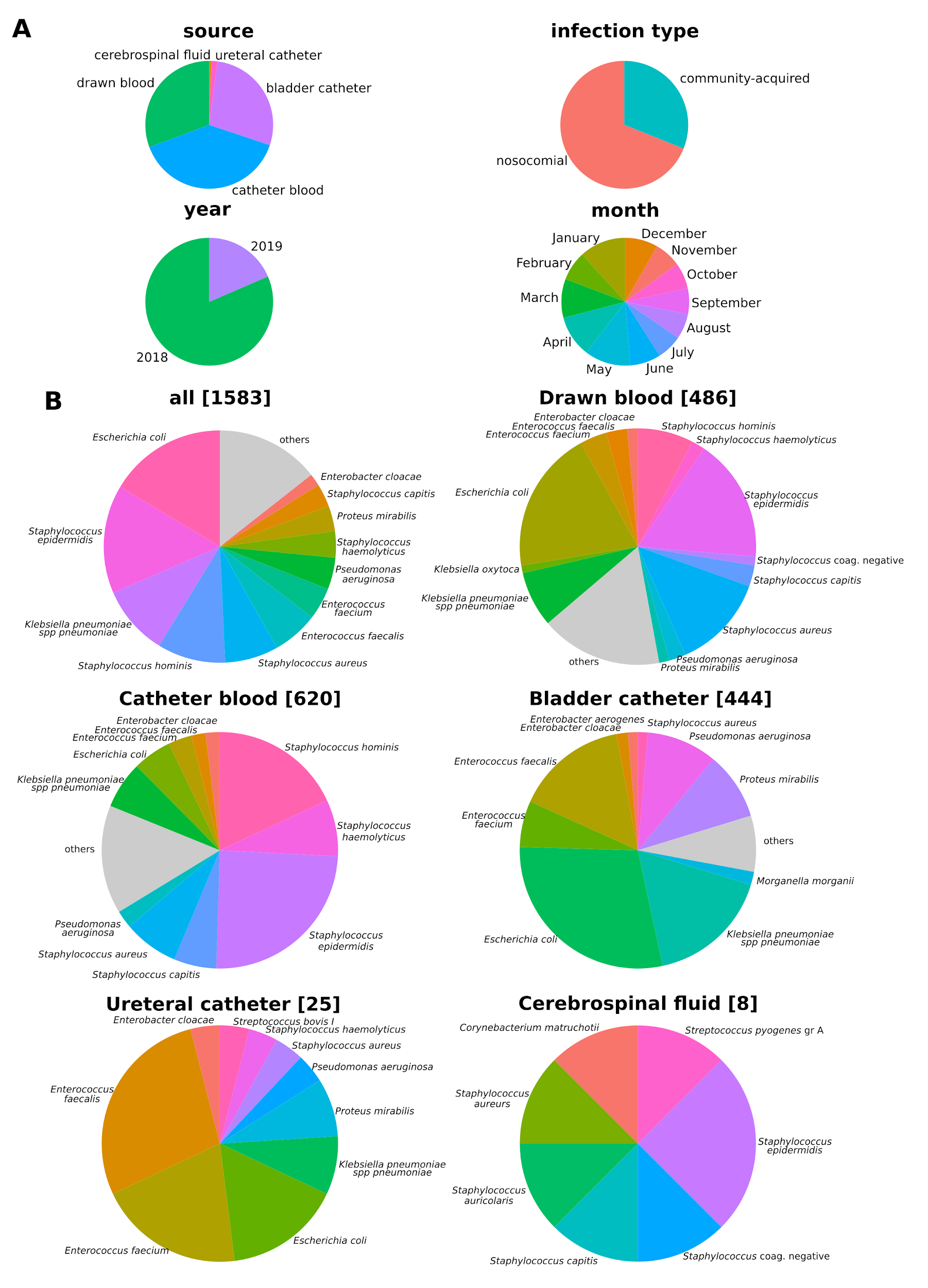


**Figure S2**

**Comparison of the composition of groups of strains isolated over the first semester of 2018 and 2019.** Strains isolated at the same time (month and year) from the same type of specimen (blood, catheter blood, bladder catheter, ureteral catheter or cerebrospinal fluid) and recognized as having the same origin (nosocomial or community-acquired) were considered as belonging to the same group. Left panel: first two coordinates of the PCoA computed on Jaccard distances among groups of isolates. Right panel: distribution of species according to the relative abundance of the microbial species in the analyzed groups of isolates. The radius of the point is proportional to the number of isolates of the corresponding species. The most abundant species are shown as colored points, grey points correspond to less abundant species.


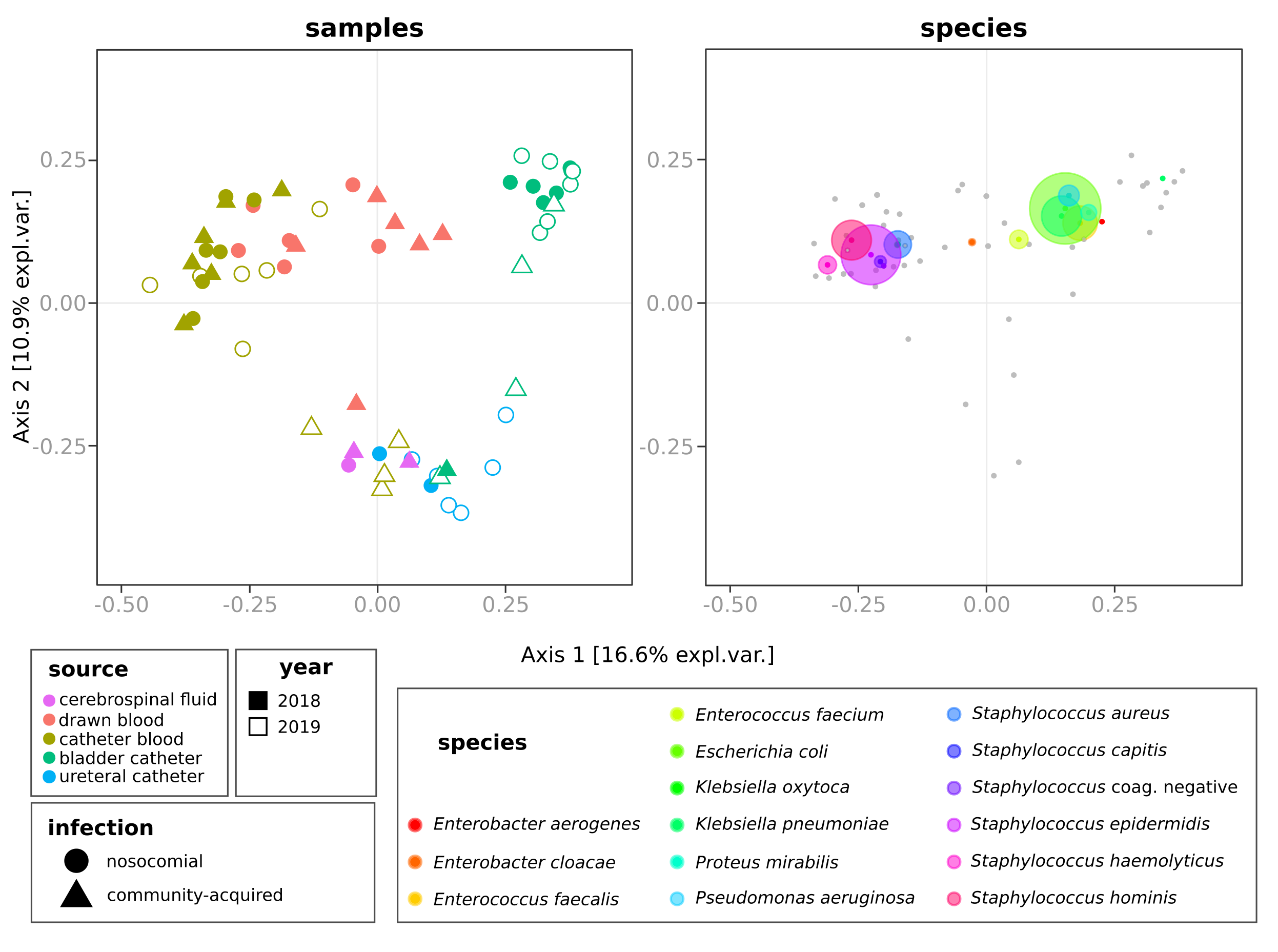


Statistics

grouping by source - permutational analysis of variance fdr= 0.001

grouping by infection - permutational analysis of variance fdr= 0.022

grouping by year - permutational analysis of variance fdr= 0.002

grouping by month - permutational analysis of variance fdr= 0.746

**Figure S3**

**Bacterial species with relative abundances differing among years (A) or sources of isolation (B) over the first semester of 2018 and 2019.** Horizontal lines indicate significant differences (Wilcoxon test fdr<0.05).


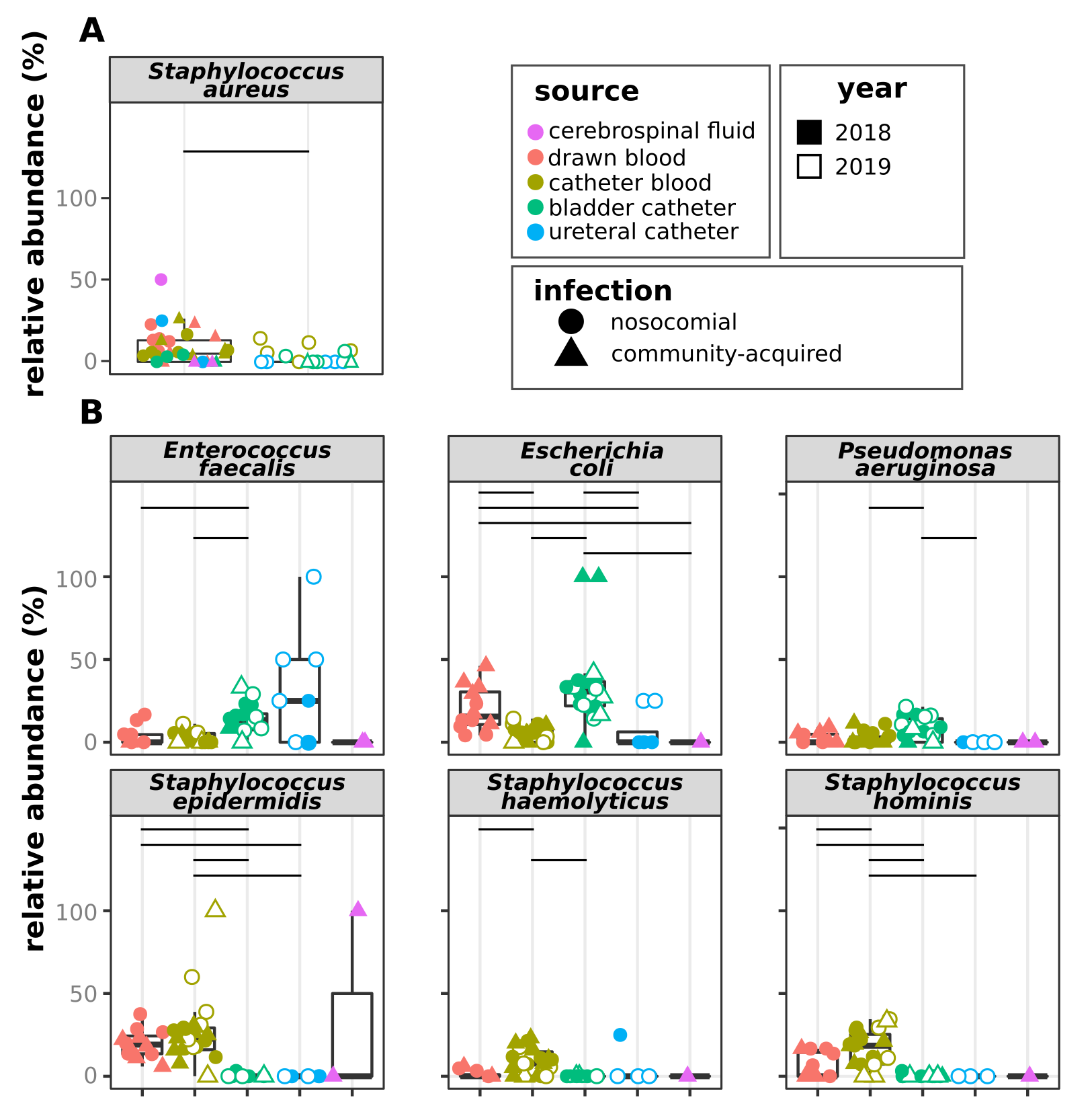


**Figure S4**

**Antibiotic resistance per species.** Each point represents a group of isolates (strains isolated from the same specimen, in the same year and month, and associated with the same type of infection). Numbers in the upper part of the plot indicate that the species shown in the boxplot has significantly different percentage of resistance compared to the species corresponding to the number (as indicated in the species labels).


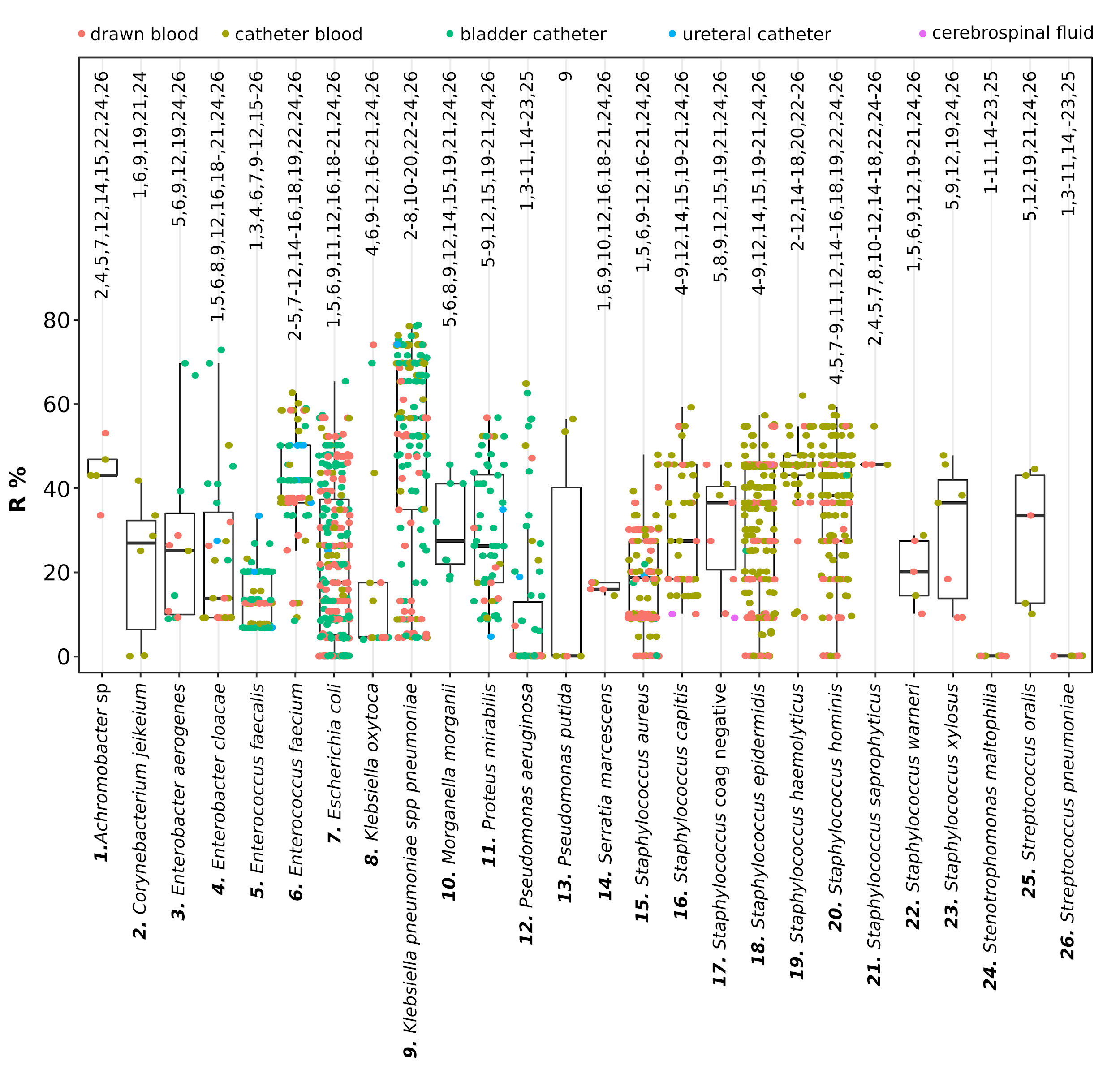

Supplement: Supplementary file 1 [file antibiotics-09-00631-s001.zip › supplementary materials/Supplementary_information_Antibiotics.docx]
